# Polymorphisms in ABC Transporter Genes and Concentrations of Mercury in Newborns – Evidence from Two Mediterranean Birth Cohorts

**DOI:** 10.1371/journal.pone.0097172

**Published:** 2014-05-15

**Authors:** Sabrina Llop, Karin Engström, Ferran Ballester, Elisa Franforte, Ayman Alhamdow, Federica Pisa, Janja Snoj Tratnik, Datja Mazej, Mario Murcia, Marisa Rebagliato, Mariona Bustamante, Jordi Sunyer, Αikaterini Sofianou-Katsoulis, Alexia Prasouli, Eleni Antonopoulou, Ioanna Antoniadou, Sheena Nakou, Fabio Barbone, Milena Horvat, Karin Broberg

**Affiliations:** 1 Foundation for the Promotion of Health and Biomedical Research in the Valencian Region, FISABIO, Valencia, Spain; 2 Spanish Consortium for Research on Epidemiology and Public Health (CIBERESP), Madrid, Spain; 3 Division of Occupational and Environmental Medicine, Lund University, Lund, Sweden; 4 School of Nursing, University of Valencia, Valencia, Spain; 5 Unit of Hygiene and Epidemiology, University of Udine, Udine, Italy; 6 Department of Environmental Sciences, Jožef Stefan Institute, Ljubljana, Slovenia; 7 Medicine Department, Jaume I University, Castelló de la Plana, Spain; 8 Centre for Research in Environmental Epidemiology (CREAL), Barcelona, Spain; 9 Hospital del Mar Research Institute (IMIM), Barcelona, Spain; 10 Center for Genomic Regulation (CRG), Barcelona, Spain; 11 Pompeu Fabra University (UPF), Barcelona, Spain; 12 Department of Social and Developmental Paediatrics Institute of Child Health, Athens, Greece; 13 Institute of Environmental Medicine, Karolinska Institutet, Stockholm, Sweden; Kagoshima University Graduate School of Medical and Dental Sciences, Japan

## Abstract

**Background:**

The genetic background may influence methylmercury (MeHg) metabolism and neurotoxicity. ATP binding cassette (ABC) transporters actively transport various xenobiotics across biological membranes.

**Objective:**

To investigate the role of ABC polymorphisms as modifiers of prenatal exposure to MeHg.

**Methods:**

The study population consisted of participants (n = 1651) in two birth cohorts, one in Italy and Greece (PHIME) and the other in Spain (INMA). Women were recruited during pregnancy in Italy and Spain, and during the perinatal period in Greece. Total mercury concentrations were measured in cord blood samples by atomic absorption spectrometry. Maternal fish intake during pregnancy was determined from questionnaires. Polymorphisms (n = 5) in the ABC genes *ABCA1*, *ABCB1*, *ABCC1* and *ABCC2* were analysed in both cohorts.

**Results:**

*ABCB1* rs2032582, *ABCC1* rs11075290, and *ABCC2* rs2273697 modified the associations between maternal fish intake and cord blood mercury concentrations. The overall interaction coefficient between rs2032582 and log2-transformed fish intake was negative for carriers of GT (β = −0.29, 95%CI −0.47, −0.12) and TT (β = −0.49, 95%CI −0.71, −0.26) versus GG, meaning that for a doubling in fish intake of the mothers, children with the rs2032582 GG genotype accumulated 35% more mercury than children with TT. For rs11075290, the interaction coefficient was negative for carriers of TC (β = −0.12, 95%CI −0.33, 0.09), and TT (β = −0.28, 95%CI −0.51, −0.06) versus CC. For rs2273697, the interaction coefficient was positive when combining GA+AA (β = 0.16, 95%CI 0.01, 0.32) versus GG.

**Conclusion:**

The ABC transporters appear to play a role in accumulation of MeHg during early development.

## Introduction

The environmental pollutant methylmercury (MeHg) originates from methylation of inorganic mercury by bacteria in aquatic systems [1]. It accumulates in the aquatic food chain and humans are mainly exposed to MeHg from fish intake. MeHg is effectively absorbed from the human gastrointestinal tract and readily crosses the placenta and blood–brain barrier [2]. Thus, there is a relationship between fish intake of pregnant women and total mercury concentrations in their newborns [3]–[6]. Exposure especially affects the nervous system. Hence, low-level exposure during pregnancy may cause impaired development in infants and children [7].

The type and the amount of fish consumed influence the maternal, and in turn the prenatal, dose of MeHg [5]. However, as with other metals [8]–[11] genetics probably influences uptake, distribution, and excretion of MeHg, and thus also the prenatal MeHg dose. The few genetic variants that have been associated with MeHg toxicokinetics identified to date have mainly been in glutathione-related genes [12]–[15].

Transporters proteins are related to the cellular intake and uptake of several types of substances; however no specific MeHg transporters have been identified yet. Mercury is capable to form complexes with small molecules such as amino acids that can mimic essential molecules recognized by transporter proteins [16]. It is important to note that a significant number of protein carriers have been identified in the placenta and it has been suggested that they may play a role in the uptake and/or efflux of MeHg complexes [17]. Therefore, genes that potentially affect MeHg metabolism include the ones encoding the superfamily of ATP binding cassette (ABC) transporters, a large and widely expressed protein family responsible for the active transport of various compounds, including drugs (e.g., anticancer agents) and xenobiotics, across biological membranes. ABCB1, ABCC1, and ABCC2 (also known as the multidrug resistance-associated proteins MDR1, MRP1 and MRP2), are the best-characterized ABC transporters. All three proteins are found at relatively high levels in the blood–brain barrier, placenta, liver, gut, and kidney and they may participate in cellular export of metal complexes [18]. ABCC1/MRP1 has been related to the extracellular transport of glutathione-conjugates in cultured astrocytes [19] and the upregulation of ABCC1 in primary mouse hepatocytes decreased the accumulation of MeHg [20]. ABCC2/MRP2 has been shown to participate in renal export of mercuric ions in rats [21], and polymorphisms in *ABCC2/MRP2* were associated with the urinary excretion of inorganic mercury in populations exposed to mercury vapour from gold mining [22]. Potential roles of ABCB1/MDR1 or the cholesterol transporter ABCA1 in mercury metabolism or toxicity remain unexplored.

The purpose of this study was to evaluate the effect of polymorphisms in ABC genes on prenatal exposure to MeHg in two Mediterranean birth cohorts.

## Methods

### 2.1 Ethics Statement

Women participating in the study signed a written informed consent form in each phase. The Ethics Committees of La Fe Hospital in Valencia, the Institut Municipal d'Assistència Sanitaria in Barcelona, the Burlo Garofolo in Trieste, the Institute of Child Health in Athens, and Lund University in Lund approved the study.

### 2.2 Study populations

Study subjects were participants in two birth cohorts from three Mediterranean countries, one cohort from the Adriatic Sea region, encompassing Italy and Greece (PHIME, Public Health Impact of long-term, low-level Mixed Element Exposure in susceptible population strata), and one cohort from Spain (INMA, in Spanish, Environment and Childhood). PHIME is a multicenter project that aims to evaluate the health effects of long-term exposure to low levels of metals. One part of PHIME addresses the effects of MeHg on the nervous system in populations from Italy, Slovenia, Greece and Croatia [23]. This study included participants from Italy and Greece; the study population consisted of 1,384 women recruited in the province of Trieste (Italy) and the Greek islands of Lesvos, Chios, Samos and Leros in the Eastern Aegean. The pregnant women eligible for recruitment were permanent residents of the study areas for at least 2 years, were at least 18 years of age, and had no absence from the study area for more than 6 weeks during pregnancy, no history of drug abuse, no serious health problems or complications of pregnancy, and no twin gestation. At recruitment, eligible women were approached for consent after their routine morphologic ultrasound scan between 20 and 22 gestational weeks (Italy, 2007–2009), or during their hospital stay for delivery (Greece, 2006–2009). Mixed umbilical cord blood mercury concentrations were analyzed in all 1,125 samples (81%) and DNA was obtained from 1,008 samples (73%).

INMA is a multicenter birth cohort study, which aims to investigate the effect of environmental exposures and diet during pregnancy on fetal and child development in different geographical areas of Spain (http:www.proyectoinma.org). Details of the INMA project and sampling procedures have been described elsewhere [24]. Briefly, pregnant women in this study were recruited during the 1^st^ trimester of pregnancy (n = 1,512) and followed until delivery (n = 1,409) in two regions of Spain, Valencia and Sabadell (2003–2007). The inclusion criteria were: an age of 16 years or over; 10–13 weeks of gestation; singleton pregnancy; intention of undergoing follow-up and delivering in the corresponding centre of reference; no impediment for communication; and no chronic disease prior to the pregnancy. Venous cord blood samples were available for analysis of mercury concentrations in 1014 (72%) newborns, and genetic analysis for 745 newborns (53%).

The final study population consisted of 1,651 mother-child pairs with complete data on mercury concentrations in cord blood and DNA available for genetic analysis.

### 2.3 Study variables

Fish intake during pregnancy was assessed using a semi-quantitative validated food frequency questionnaire (FFQ) in both studies [4], [5]. In the PHIME study, the FFQ was administered during the third trimester of pregnancy (30–32^nd^ week) in Italy and 3–6 months after delivery in Greece. The questionnaire in Italy covered until the third trimester of pregnancy and the questionnaire in Greece the whole pregnancy period. The FFQ included questions about the following different types of seafood: fresh fish, crustaceans, molluscs, tuna, mackerel, and sardines in oil. The frequencies in this FFQ ranged from “never” to “3 or more times per day”.

In the INMA study, the FFQ was administered during the first (10–14^th^ week) and the third (28–32^nd^ week) trimesters of pregnancy and it covered average seafood intake from the last menstrual period until the third trimester of pregnancy. The FFQ included questions about intake of different types of seafood:, lean fish, large oily fish, small oily fish, smoked or dry fish, mixed fried fish, canned tuna, canned sardines or mackerel, crustaceans, bivalves, cephalopods and processed fish. The frequencies in each fish category ranged from “never” or “less than once per month” to “6 times or more per day”. For the present study, we used the average of the intakes from the FFQ at each time point.

The average daily intake for each fish item was added up to compute the total fish intake per week in the three countries (weekly servings). In order to have a comparable estimation of fish intake during pregnancy between countries, frequencies were homogenized and the categories of smoked or dry and processed fish in the INMA study were excluded.

Information about socio-demographic characteristics was also obtained through questionnaires at the same time points as the FFQ in the three countries. The covariates used in this study were: maternal age at delivery, maternal and paternal educational level, maternal employment status during pregnancy, country of birth, parity, and child's sex. The number of maternal amalgam fillings was also obtained for the INMA participants.

### 2.4 Mercury exposure

Total mercury concentrations were measured in cord whole blood samples as a measure of the child's exposure to MeHg during pregnancy. The analytical procedure has been described elsewhere [4], [5]. The analysis of the PHIME samples was carried out in the laboratory of the Department of Environmental Sciences at the Jožef Stefan Institute in Ljubljana, Slovenia and the INMA samples in the Public Health Laboratory in Alava (LSPPV), Basque Country, Spain. Briefly, total mercury was measured by thermal decomposition, amalgamation, and atomic absorption spectrometry. PHIME used a Direct Mercury Analyser (Milestone, USA). The INMA study used an AMA-254 (LECO Corporation, St. Joseph, Michigan). The limit of quantification (LOQ) of the procedure was 0.07 ppb in PHIME and 2.0 ppb in INMA. For measurements below the LOQ in the INMA study (n = 34) we used the approximation LOQ/√2.

In the PHIME cohort, MeHg was measured in cord blood (n = 221 in Italy and n = 196 in Greece) for all mothers with total Hg in hair exceeding 1 µg/g. The analytical procedure is found in the Supplemental Material Part S1 in File S1.

For quality control in both laboratories the reference material “Seronorm Trace Elements in Whole Blood” was used to check the accuracy of the results (LOT no. MR4206 at the Jožef Stefan Institute, and LOT no: 0503109 and no. 201605 at the LSPPV). The average recovery for reference materials in LSPPV was 93% (85% to 104%) and in Jožef Stefan Institute was 95% (from 90 to 100%).

Additionally, both laboratories participated in inter-laboratory comparisons. The Jožef Stefan Institute laboratory participated in three inter-laboratory comparisons organised within the PHIME project (Mazej et al. 2008). Since 2009, LSPPV has participated (3 times per year) in inter-laboratory comparisons organized by the New York State Department of Health in the Wadsworth Center. Both laboratories obtained satisfactory results.

### 2.5 Genetic analysis

This is a candidate gene approach where we have analysed SNPs in main ABC transporters of potential importance for MeHg retention. We selected SNPs based on functional impact according to the literature, potential functional impact according to position and type of SNP (specifically, non-synonymous SNPs that may affect the protein structure/enzyme activity or 5′ SNPs at putative promoter sites that can affect gene expression) [25]; or tagSNPs that capture as much of the genetic variation within a gene segment as possible due to linkage disequilibrium (LD) with other SNPs. TagSNPs were selected according to HapMap data [26] for CEU (CEPH, Utah residents with ancestry from northern and western Europe). Here 5 SNPs were analyzed in genes coding for 4 different ABC transporters: rs3905000 (*ABCA1*), rs3213619 and rs2032582 (*ABCB1*), rs11075290 (*ABCC1*), and rs2273697 (*ABCC2*). The SNPs were analyzed by mass spectrometry in the PHIME cohort and by beadchip analysis in the INMA cohort.

In the PHIME cohort, DNA was extracted from cord blood using the Qiagen DNA Blood Mini kit (Qiagen, Hilden, Germany). SNPs were genotyped by using the iPLEX Gold assay on the MassARRAY platform (Sequenom, San Diego, CA, USA). Five percent of the samples were re-analyzed for quality control purposes with perfect agreement between original and repeat genotyping runs for all SNPs. The following quality control thresholds were applied: population call rate >60% (n = 8 individuals from Greece and n = 12 from Italy were excluded), SNP call rate >90% and MAF>5% (n = 36 SNPs from Greece and n = 34 from Italy were excluded). The final genetic data consisted of 1009 subjects (656 from Italy and 352 from Greece).

In the INMA cohort, DNA was obtained from cord blood using the Chemagen protocol (Baesweiler, Germany) at the Spanish National Genotyping Centre (CEGEN). Genotyping was performed using the HumanOmni1-Quad Beadchip (lllumina, San Diego, CA, USA) at CEGEN. Genotype calling was done using the GeneTrain2.0 algorithm based on HapMap clusters implemented in the GenomeStudio Illumina software. The following initial quality control thresholds were applied: sample call rate>98% and/or logRRatio SD<0.3 (n = 4 were excluded in Valencia). Then, sex, relatedness (excluded: one duplicated sample and the younger brother of each of two brother-pairs detected in Sabadell), heterozygosity and population stratification were checked. Principal component analysis (PCA) showed that there was no population stratification in the cohort. Genetic variants were filtered for single nucleotide polymorphism (SNP) call rate>95%, and MAF>1%. The final genetic data set consisted of 748 subjects from Sabadell (N = 399) and Valencia (N = 349). From this data set we selected the 5 ABC transporter gene SNPs analysed in the PHIME study (see above).

### 2.6 Statistical analysis

We calculated the log2 of maternal fish intake and of mercury concentrations in cord blood to improve linearity. When the frequency of a homozygote genotype was low (<10%), this group was pooled with the heterozygotes. One of the SNPs (rs3213619) was not in Hardy Weinberg equilibrium (p Chi^2^<0.05) in Greece; it was therefore excluded from further analysis. We analysed mercury concentrations (geometric means and 95% confidence intervals (CI)) in relation to genotype by the Kruskal-Wallis test.

To analyse the modification of ABC transporter SNPs upon the relation between fish intake and cord blood total Hg concentrations we performed linear regression analyses. Linear regression models were adjusted for variables with a p-value<0.1 in the Likelihood Ratio test using a backward procedure. We performed a sensitivity analysis replacing total mercury concentrations by MeHg for a subsample of PHIME participants where MeHg was measured.

We performed an analysis in each country and combined estimation through meta-analysis of the interaction between fish intake during pregnancy and genotype. In order to examine whether there was heterogeneity, estimates by country it was quantified with the I-squared measure (I^2^) [27] under the fixed-effect hypothesis and, if heterogeneity was detected (I^2^>50%), we applied the “random effect model”. As the heterogeneity was low a pooled analysis was performed to evaluate the genetic effect modification in all individuals using a common model and adjusting by cohort. We stratified the study population according to genotype and evaluated the association between fish intake and cord blood mercury concentrations by linear regression for each genotype separately.

Due to different molecular techniques used in the two birth cohorts, three alleles were determined for rs2032582 (*ABCB1*) in PHIME (G, T, A), and two in INMA (G, T). We assumed that the A allele detected by the Illumina chip (INMA) was classified as other alleles: GA as GT, AA as TT, and AT as TT. In order to obtain comparable genetic information among the countries, we reorganized the genotypes in PHIME as follows: the GA genotype was GT, the AA and AT genotypes were TT. Further, in a sensitivity analysis we also excluded the individuals with the A allele in PHIME from the analysis. The analyses were carried out by using the Stata version 11 statistical package (StataCorp LP, College Station, Texas).

## Results

Questionnaire and genetic data and mercury concentrations were obtained for 964 mother-child pairs (593 from Italy and 371 from Greece) in the PHIME cohort, 70% of the original population; and 687 mother-child pairs from the INMA cohort, 49% of the original population. In PHIME, no significant differences were found between the study population and the original cohort. In INMA, differences found between the study population and the original cohort were regarding maternal age and the country of birth (women who participated in this study were a bit older and the percentage of foreign women was lower than in the original cohort).

Spanish women consumed fish more frequently (mean: 6.3±2.6 weekly servings) than Greek (mean: 3.2±2.3) and Italian women (mean: 2.5±1.6) (Table 1). Spanish newborns also had higher cord blood mercury concentrations (geometric mean: 8.2 µg/L, 95%CI 7.7, 8.7) than the Greek (5.4 µg/L, 95%CI 4.9, 5.8) and Italian newborns (3.8 µg/L, 95%CI 3.6, 4.1). The Spearman correlation coefficients between fish intake during pregnancy and cord blood mercury concentrations were 0.42 in Italy, 0.26 in Spain, and 0.18 in Greece. The correlation between cord blood mercury concentrations and number of dental amalgam fillings among the Spanish women was r = 0.02. There were some differences in characteristics (educational level, age of the mothers, percentage of foreign mothers, and percentage of women who worked during pregnancy) between the countries (Table 1).

**Table 1:** Characteristics of the study populations.

		Italy	Greece	Spain
Characteristic		N (%)	N (%)	N (%)
Maternal educational level	Up to primary	5 (0.9)	40 (13.2)	205 (29.9)
	Secondary	365 (66.1)	197 (65.0)	298 (43.4)
	University	182 (33.0)	66 (21.8)	183 (26.7)
Paternal educational level	Up to primary	7 (1.3)	44 (14.6)	280 (41.0)
	Secondary	402 (73.8)	204 (67.8)	284 (41.6)
	University	136 (25.0)	53 (17.6)	119 (17.4)
Maternal age at delivery	<25	20 (3.7)	49 (16.8)	30 (4.4)
	25–29	82 (15.2)	110 (37.8)	211 (30.8)
	30–34	232 (43.0)	83 (28.5)	304 (44.3)
	> = 35	205 (38.0)	49 (16.8)	141 (20.6)
Country of birth	National	506 (92.0)	281 (93.0)	664 (96.8)
	Other	44 (8.0)	21 (7.0)	22 (3.2)
Maternal employment during pregnancy	No	77 (13.9)	146 (48.0)	88 (12.8)
	Yes	476 (86.1)	158 (52.0)	598 (87.2)
Parity	0	311 (45.0)	116 (38.0)	425 (57.1)
	1	241 (34.9)	106 (34.8)	280 (37.6)
	≥2	139 (20.1)	83 (27.2)	39 (5.2)
Sex of the children	Male	288 (52.5)	138 (45.7)	362 (52.8)
	Female	261 (47.5)	164 (54.3)	324 (47.2)
Fish intake during pregnancy (weekly servings)^a^		2.5±1.6	3.2±2.3	6.2±2.6
Hg (µg/L)^b^		3.8 (3.6, 4.1)	5.4 (4.9, 5.8)	8.2 (7.7, 8.7)

a Mean and standard deviation.

b Geometric mean and 95% confidence intervals.

The allele frequencies were similar between the countries (Table 2). The minor allele was the same in each country for all SNPs, except for rs11075290 in *ABCC1* in Greece; however, the same reference allele was used in all analyses for this SNP. Some differences in mercury concentrations between the genotypes were found. Newborns from Italy with TT genotype for *ABCB1* rs2032582 had higher cord blood mercury concentrations (4.2 µg/L) than newborns with GT (4.0 µg/L) and GG (3.2 µg/L) genotypes. Spanish newborns with TC genotype for *ABCC1* rs1107529 had higher concentrations (geometric mean = 8.8 µg/L) than CC (8.0 µg/L) and TT (7.5 µg/L) genotypes. Italian newborns with GG and AG genotype for *ABCC2* rs2273697 had similar cord blood mercury concentrations (3.9 µg/L) that were higher than newborns with AA (2.9 µg/L).

**Table 2:** Genetic characteristics and cord blood mercury concentrations (geometric means and 95% confidence intervals) according to genotype.

			Italy					Greece					Spain				
SNPs	Gene	Genotype^a^	N (%)	HWE p^c^	MAF^b^	HgGM (95%CI)	p-value^d^	N (%)	HWE p^c^	MAF^b^	HgGM (95%CI)	p-value^d^	N (%)	HWE p^c^	MAF^b^	HgGM (95%CI)	p-dvalue
rs3905000	*ABCA1*	GG	434 (73.4)	0.272	0.15	3.9 (3.6, 4.2)	0.808	283 (76.5)	0.377	0.13	5.3 (4.8, 5.9)	0.859	475 (69.4)	0.649	0.17	8.1 (7.6, 8.7)	0.550
Intronic (Chr 9)		AG	141 (23.9)			3.9 (3.4, 4.4)		79 (21.4)			5.6 (4.7, 6.6)		188 (27.5)			8.3 (7.3, 9.4)	
		AA	16 (2.7)			3.3 (1.9, 5.8)		8 (2.2)			6.9 (3.5, 13.8)		21 (3.1)			10.1 (6.8, 15.0)	
rs2032582	*ABCB1*	GG	155 (26.9)	0.163	0.47	3.2 (2.8, 3.7)	0.018	128 (36.0)	0.783	0.40	5.4 (4.6, 6.3)	0.617	226 (33.0)	0.522	0.42	8.8 (7.9, 9.9)	0.202
Non-synonymous (Chr 7)		GT	304 (52.7)			4.0 (3.7, 4.4)		173 (48.6)			5.5 (4.9, 6.3)		342 (49.9)			8.1 (7.4, 8.8)	
		TT	118 (20.5)			4.2 (3.6, 4.9)		55 (15.4)			4.9 (3.9, 6.1)		117 (17.1)			7.5 (6.5, 8.7)	
rs3213619	*ABCB1*	AA	578 (97.4)	0.616	0.02	3.9 (3.6, 4.1)	0.336	347 (93.7)	<0.001	0.04	5.4 (4.9, 5.9)	0.728	624 (91.0)	0.663	0.05	8.2 (7.7, 8.7)	0.838
5′ UTR (Chr 7)		AG	14 (2.3)			3.1 (1.8, 5.3)		20 (5.4)			5.5 (3.8, 7.8)		60 (8.7)			8.3 (6.6, 10.4)	
		GG	0					3 (0.8)			7.0 (0.5, 93.7)		2 (0.3)			13.5 (8.4, 21.6)	
rs11075290	*ABCC1*	CC	115 (19.9)	0.346	0.44	4.4 (3.8, 5.3)	0.046	94 (25.8)	0.996	0.49	5.9 (5.0, 7.0)	0.571	108 (16.0)	0.908	0.40	8.0 (6.9, 9.2)	0.014
Intronic (Chr 16)		TC	273 (47.2)			3.5 (3.2, 3.9)		182 (50.0)			5.2 (4.6, 5.9)		325 (48.3)			8.8 (8.1, 9.7)	
		TT	190 (32.9)			3.9 (3.5, 4.4)		88 (24.2)			5.4 (4.5, 6.4)		240 (35.7)			7.5 (6.8, 8.3)	
rs2273697	*ABCC2*	GG	360 (61.3)	0.618	0.21	3.9 (3.5, 4.2)	0.078	216 (58.4)	0.723	0.23	5.4 (4.8, 6.1)	0.919	446 (65.0)	0.226	0.20	8.2 (7.6, 8.8)	0.292
Non-synonymous (Chr 10)		AG	202 (34.4)			3.9 (3.5, 4.5)		135 (36.5)			5.4 (4.7, 6.2)		208 (30.3)			7.9 (7.1, 8.9)	
		AA	25 (4.3)			2.9 (2.2, 3.9)		19 (5.1)			5.1 (3.6, 7.2)		32 (4.7)			10.3 (7.9, 13.3)	

a Reference genotype is the homozygote for the ancestral allele.

b MAF: minor allele frequency.

c HWE p: p-value for the Chi2-test of Hardy-Weinberg equilibrium.

d p-value obtained by Kruskal-Wallis test.

GM: geometric mean.

**Figure 1:**
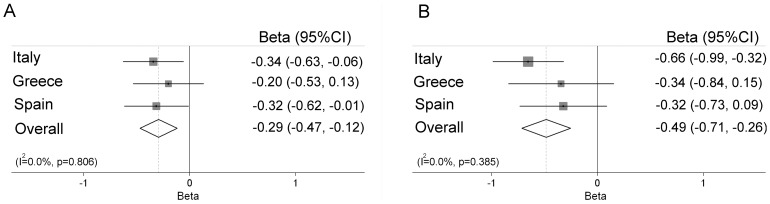
Meta-analysis of the interaction between fish intake and the SNP rs2032582 in *ABCB1* on cord blood mercury concentrations, presented as beta values for carriers of GT (A) and TT (B) vs. GG genotype.

**Figure 2:**
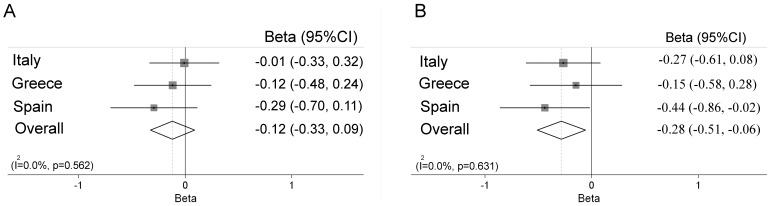
Meta-analysis of the interaction between fish intake and rs11075290 in *ABCC1* on cord blood mercury concentrations, presented as beta values for carriers of TC (A) and TT (B) vs. CC genotype.

**Figure 3:**
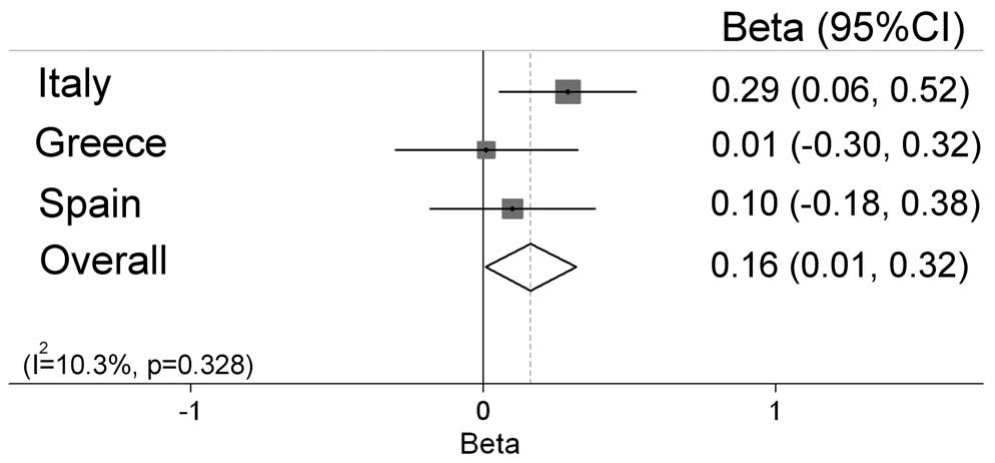
Meta-analysis of the interaction between fish intake and the SNP rs2273697 in *ABCC2* on cord blood mercury concentrations, presented as beta values for carriers of AG or AA genotypes vs. GG genotype.

In a meta-analysis, we found that *ABCB1* rs2032582, *ABCC1* rs11075290 and *ABCC2* rs2273697 showed a genetic effect on cord blood mercury concentrations, in the same direction in cohorts from the three countries (Figures 1–3). No heterogeneity was found in the effect estimates (beta values) for the interaction between fish intake and genotype for rs2032582, rs11075290, and rs2273697 on mercury concentrations comparing the different countries. For *ABCB1* rs2032582 the overall coefficient was negative for both carriers of GT (β = −0.29, 95%CI −0.47, −0.12) and TT (β = −0.49, 95%CI −0.71, −0.26) vs. GG carriers (Figure 1, A and B) meaning that for a doubling in fish intake of the mothers, children with the rs2032582 GG genotype accumulated 30% more mercury than children with TT. For *ABCC1* rs11075290, the overall coefficient was negative for the TC (β = −0.12, 95%CI −0.33, 0.09), but stronger for the TT (β = −0.28, 95%CI −0.51, −0.06) carriers compared to CC (Figure 2 A and B). Finally, for *ABCC2* rs2273697 the overall coefficient for the interaction between genotype and the fish intake was positive for GA+AA vs. GG carriers (β = 0.16, 95%CI 0.01, 0.32; Figure 3). In the meta-analysis for the overall coefficient was not statistically significant (Figure S1 in File S1).

The same models were then performed in a pooled data analysis (Table 3). Statistically significant interactions were found for fish intake and *ABCB1* rs2032582, *ABCC1* rs11075290, and *ABCC2* rs2273697 and the effect estimates for the genotypes showed a similar direction as in the meta-analysis. Adjustment for influential variables did not change the results (Table 3). We performed a sensitivity analysis by excluding individuals from Italy and Greece with the A allele for the SNP rs2032582 (n = 26 and n = 19, respectively) and this did only affect the effect estimates marginally and not the significance level (not in table).

**Table 3:** A pooled analysis of associations between a doubling in fish intake during pregnancy and cord blood mercury concentrations.

	Unadjusted models^a^^,^ b		Adjusted models^c^^,^ d	
	beta	p-value	beta	p-value
**All newborns**	0.52 (0.44, 0.59)		0.50 (0.41, 0.59)	
**rs3905000 (** ***ABCA1*** **)**				
GG	0.51 (0.42, 0.59)	0.668	0.52 (0.37, 0.68)	0.721
GA+AA	0.55 (0.40, 0.70)		0.53 (0.37, 0.68)	
**rs2032582 (** ***ABCB1*** **)**				
GG	0.74 (0.60, 0.87)	<0.001	0.70 (0.56, 0.85)	<0.001
GT	0.45 (0.35, 0.56)		0.45 (0.34, 0.56)	
TT	0.25 (0.07, 0.43)		0.27 (0.08, 0.46)	
**rs11075290 (** ***ABCC1*** **)**				
CC	0.65 (0.49, 0.81)	0.025	0.63 (0.47, 0.80)	0.012
TC	0.54 (0.42, 0.65)		0.55 (0.43, 0.66)	
TT	0.39 (0.26, 0.52)		0.36 (0.22, 0.50)	
**rs2273697 (** ***ABCC2*** **)**				
GG	0.45 (0.35, 0.54)	0.048	0.45 (0.35, 0.55)	0.038
GA+AA	0.63 (0.51, 0.75)		0.62 (0.50, 0.74)	

p-value for the interaction term between log2-fish intake and genotype.

a Unadjusted model: Log2 Hg∼α+β Log2fish intake +γ cohort.

b Adjusted by cohort.

c Adjusted model: Log2 Hg∼α+β Log2fish intake +γ cohort +δ covariates.

d Adjusted by cohort, maternal age, maternal educational level, maternal employment status, country of birth, parity, and weeks of gestation.

We also attempted to eliminate other sources of mercury by accounting for the effect of dental amalgam fillings. When we included the number of dental amalgam fillings into the models for Spain, the effect of fish consumption on mercury concentrations was marginally lower for all SNPs, but the interaction term were very similar (data not shown). Cord blood MeHg concentrations were obtained for a subsample of the study population from Italy and Greece. The Spearman correlation between total Hg and MeHg was high in both countries (r_S_>0.95). The β-coefficients and interaction p-values in the linear regression analysis were very similar if using either total Hg or MeHg in the models (data not shown).

## Discussion

In this study, we examined two large Mediterranean birth cohorts and show that the association between maternal fish intake and mercury in cord blood has different magnitudes depending on the child's genotype for ABC transporters *ABCB1*, *ABCC1*, and *ABCC2*. These findings strengthen the hypothesis that ABC transporters play a role in mercury transport across the placenta and accumulation of MeHg during early development. As these genes appear to influence MeHg internal dose, they might offset MeHg neurotoxicity. The risk allele frequencies in this Mediterranean study population were similar (*ABCB1* rs2032582) or somewhat higher (*ABCC2* rs2273697) than in a Japanese population (0.45 and 0.13, respectively) (NCBI, 2006: http://www.ncbi.nlm.nih.gov/SNP), which suggests that the results of this study might also be relevant for other populations with high fish consumption.

Genetic variation in *ABCC2* has recently been shown to be related to the metabolism of inorganic mercury [22]. The GA+AA genotypes of rs2273697 were associated with lower urinary mercury concentrations than the GG in two populations of gold miners exposed to mercury vapour. This SNP was associated with cord blood mercury in our study populations, but in the opposite direction; the association between fish intake and total mercury concentrations was the highest among the newborns with the GG+AA genotypes. The reason for these contrasting findings probably lies in the differences in orientation of ABCC2/MRP2 in different tissues. ABCC2/MRP2 transporters are found on the epithelial cells of the proximal tubules in kidneys, where they mediate the export of certain xenobiotics from blood to urine [17]. High levels of ABCC2/MRP2 are found in the apical syncytiotrophoblast membrane of the placenta, where it is likely responsible for preventing the passage of conjugated metabolites of drugs and toxicants from maternal to fetal circulation [28]. Thus, an ineffective ABCC2/MRP2 transporter would result in lower inorganic urinary mercury concentrations and higher cord blood MeHg concentrations.

We also found associations for *ABCB1* rs2032582 respectively *ABCC1* rs11075290 and MeHg concentrations in the pooled data analysis. Here the coefficients obtained in the meta-analysis for both SNPs suggest that the association is stronger among carriers of two variant alleles than in carriers of one allele. Moreover, the effects seem to be dose-dependent, following a pattern similar to mercury concentrations (Spain>Greece>Italy). We speculate that T carriers of *ABCB1* rs2032582 have higher capacity of exporting MeHg from the placenta. The role of ABCB1 transporters present in apical syncytiotrophoblast has been well defined with respect of the protection of the fetus from exposure to drugs and xenobiotics [29]. Enhanced expression of the ABCB1 protein was associated to the reduction of pulmonary mercury in rats exposed to elemental mercury [30] and ABCB1 has been involved in the excretion of glutathione conjugated with other metals such as arsenic in knockout mice studies [17], [31]. We further suggest that *ABCC1* rs11075290 T carriers have higher gene expression. This allele abolishes a potential CpG site and is located in a regulatory region (http://www.ensembl.org) [30]. ABCC1 prevents toxicants to the fetal blood stream and higher expression of the gene would result in less MeHg to fetal blood. It has been observed that upregulation of ABCC1/MRP1 in primary mouse hepatocytes decreased methylmercury accumulation [20].

This study had several strengths. The study population was large and derived from three different Mediterranean countries, with different fish consumption customs and a wide range of cord blood mercury concentrations, from 0.1 µg/L in Italy to 66 µg/L in Spain. We controlled that the majority of the total mercury in the cord blood derived from MeHg; in INMA we also adjusted for amalgam fillings and in PHIME we did a sensitivity analysis using MeHg measured in cord blood. In both analyses, we obtained very similar results to those obtained when using total mercury in the models. The genetic background was similar in the three countries, as indicated by the lack of heterogeneity in the meta-analysis, which justified a pooled data analysis, thus increasing the sample size and the possibility of obtaining associations.

Nevertheless, some limitations were identified that may have contributed to the uncertainty of the conclusions drawn. The analytical method used to measure the cord blood mercury concentrations was different between the cohorts. The LOQ was 30 times higher in INMA than in PHIME (2 ng/g vs. 0.07 ng/g, respectively); however, only 34 samples in the INMA study had concentrations below the LOQ. A sensitivity analysis was performed removing these 34 individual and the results obtained were the same as with the whole study population. The pattern of fish consumption (Spain>Greece>Italy) observed in this study was in accordance with previous studies [32]. However, differences in the fish intake variable were identified which limit the comparability between the cohorts. The timing of food frequency questionnaires (FFQ) was not the same in the countries. Indeed, the correlation between the fish intake and the cord blood mercury concentrations was lower in Greece, where the FFQ was administered 3–6 months after delivery, rather than during pregnancy. The fish categories included in the FFQ were also different among the countries, but we mitigated this by using an estimation of total fish intake for all countries. The methods of genotyping were different between the cohorts and this resulted in differences in detection for one of the SNPs (rs2032582, *ABCB1*), which is triallelic. We assumed that the A allele was detected as T in the INMA cohort and when merging the genotypes accordingly in the PHIME cohort the allelic percentages were very similar between the study populations. Additionally the sensitivity analysis excluding individuals with the A allele performed showed the robustness of this assumption.

Despite these limitations, we identified a modifying effect of some polymorphisms in ABC genes on the association between fish intake during pregnancy and cord blood mercury concentrations. The associations reported were robust and consistent in the different analyses.

## Conclusions

In conclusion, we found that *ABCB1* rs2032582, *ABCC1* rs11075290, and *ABCC2* rs2273697 were associated with mercury accumulation in the fetus. Children with the genotype GG of rs2032582, CC of rs11075290, and GA or AA of rs 2273697 showed a stronger association between maternal fish intake and cord blood mercury concentrations. This study not only provides basic knowledge for gene-environment interactions, a key element for determining susceptibility, but also explains specifically for MeHg why some individuals accumulate more of MeHg from fish, and possibly, suffer from more toxic effects.

## Supporting Information

File S1 **This file contains Figure S1 and Supplementary Materials Part S1.** Part S1, Analytical procedure of MeHg in cord blood samples from Italy and Greece. Figure S1, Meta-analysis of the interaction (presented as beta values for AG+AA vs. GG genotypes) between fish intake and the SNP rs3905000 in *ABCA1* on cord blood mercury concentrations.
(DOC)
