# Supplementary material for: Polymorphisms in ABC Transporter Genes and Concentrations of Mercury in Newborns – Evidence from Two Mediterranean Birth Cohorts
Source: PLoS One. 2014 May 15;9(5):e97172. doi: 10.1371/journal.pone.0097172 (PMC4022503; doi:10.1371/journal.pone.0097172)
Supplement: File S1 — This file contains Figure S1 and Supplementary Materials Part S1. Part S1, Analytical procedure of MeHg in cord blood samples from Italy and Greece. Figure S1, Meta-analysis of the interaction (presented as beta values for AG+AA vs. GG genotypes) between fish intake and the SNP rs3905000 in ABCA1 on cord blood mercury concentrations. (DOC) [file pone.0097172.s001.doc]

**Part S1: Analytical procedure of MeHg**

About 200 mg of blood sample was weighed directly in a 30 ml screw capped Teflon vial to which 6 ml of a mixture of 5% H_2_SO_4_ (Merck, Germany, suprapur), 18% KBr (Merck, Germany, p.a.) and 1.0 ml of a 1 M solutions of CuSO_4_ (Merck, Germany, p.a.) were added. After shaking the vials vigorously, solvent extraction and aqueous phase ethylation were performed. The ethylated MeHg as ethylmercury was purged onto a Tenax trap for 15 min with nitrogen gas. The Tenax trap was then connected to a flow of argon and MeHg was thermally desorbed (180°C) onto an isothermal GC column. Hg species were converted to Hg by pyrolysis at 600°C and measured by a cold vapour atomic fluorescence detector (CV AFS). The procedure has been described in detail elsewhere (Horvat et al., 1993; Liang et al., 1994).

The accuracy of the results for MeHg was checked by analysing RM lyophilised whole human blood PT-WB1 obtained from a non-exposed population. MeHg in PT-WB1 was determined by the laboratories participating in the PHIME interlaboratory comparison. The determined value (6.2 ng/g) was in good agreement with the assigned value (6.3±0.5 ng/g). The LOD of the method for MeHg determination in blood calculated on the basis of three times the standard deviation of the blanks was 0.02 ng/g blood, while the LOQ calculated as ten times the standard deviation of the blanks was 0.07 ng/g blood.

*References*:

Horvat M, Liang L, Bloom NS (1993). Comparison of distillation with other current isolation methods for the determination of methyl mercury compounds in low level environmental samples, Part 2. Water Anal Chim Acta 282:153–168.

Liang L, Horvat M, Bloom NS (1994). An improved method for speciation of mercury by aqueous phase etylation, room temperature precollection, GC separation and CV AFS detection. Talenta 3: 371–379.


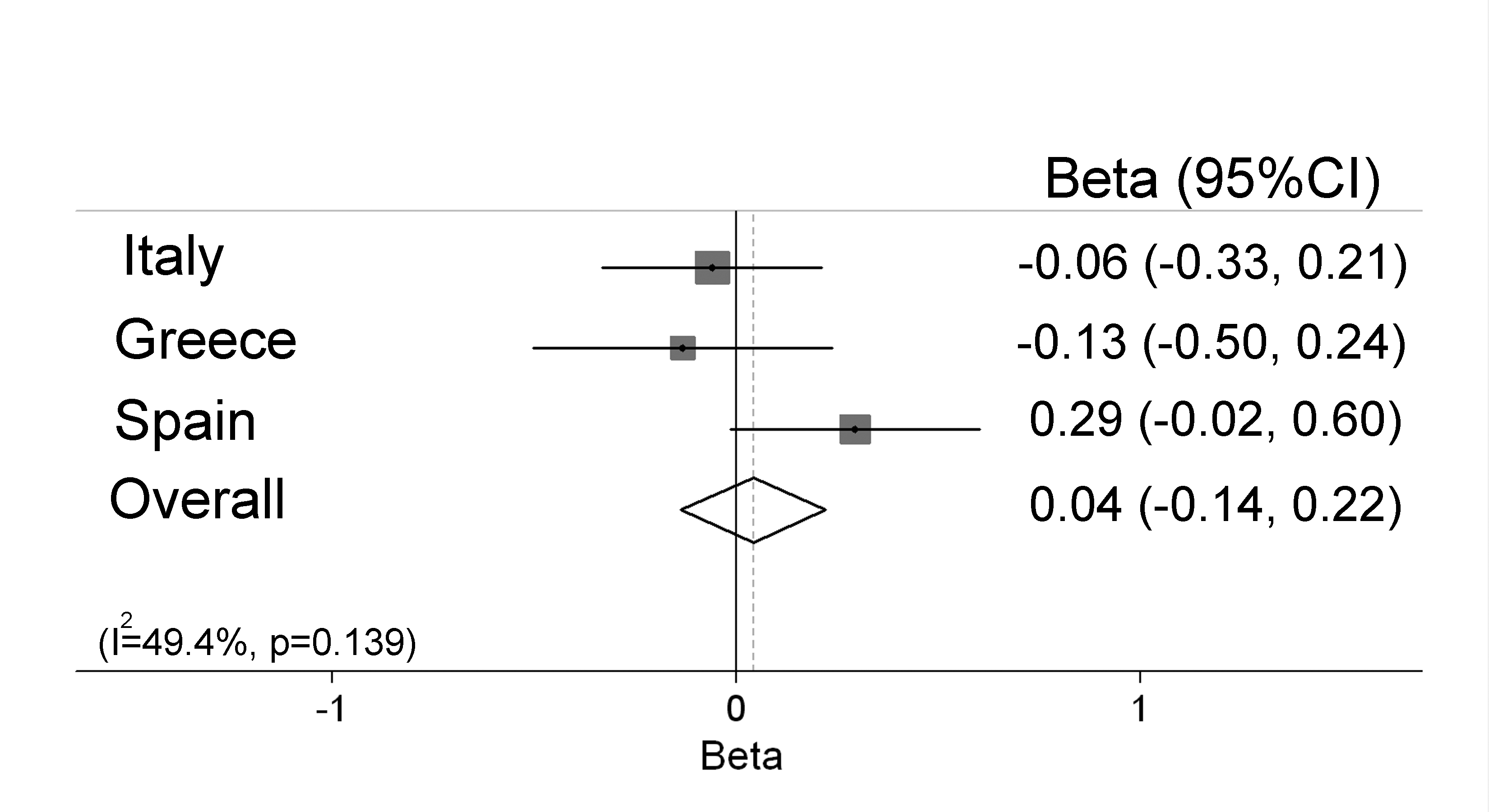


**Figure S1**. Meta-analysis of the interaction (presented as beta values for AG+AA vs. GG genotypes) between fish intake and the SNP rs3905000 in *ABCA1* on cord blood mercury concentrations.
